# Differentiated Thyroid Carcinoma in Pediatric Age: Genetic and Clinical Scenario

**DOI:** 10.3389/fendo.2019.00552

**Published:** 2019-08-07

**Authors:** Francesca Galuppini, Federica Vianello, Simona Censi, Susi Barollo, Loris Bertazza, Sofia Carducci, Chiara Colato, Jacopo Manso, Massimo Rugge, Maurizio Iacobone, Sara Watutantrige Fernando, Gianmaria Pennelli, Caterina Mian

**Affiliations:** ^1^Pathology Unit, Department of Medicine (DIMED), Padova University Hospital, Padova, Italy; ^2^Department of Women's and Children's Health, Padova University Hospital, Padova, Italy; ^3^Department of Radiotherapy, Istituto Oncologico del Veneto, IOV-IRCCS, Padova, Italy; ^4^Endocrinology Unit, Department of Medicine (DIMED), Padova University Hospital, Padova, Italy; ^5^Pathology Section, Department of Pathology and Diagnostics, University of Verona, Verona, Italy; ^6^Endocrine Surgery Unit, Department of Surgical, Oncological and Gastroenterological Sciences (DiSCOG), Padova University Hospital, Padova, Italy; ^7^Familial Cancer Clinic, Istituto Oncologico Veneto IOV-IRCCS, Padova, Italy

**Keywords:** childhood, thyroid cancer, BRAF, TERT, RAS

## Abstract

**Introduction:** Follicular-derived differentiated thyroid carcinoma (DTC) is the most common endocrine and epithelial malignancy in children. The differences in the clinical and pathological features of pediatric vs. adult DTC could relate to a different genetic profile. Few studies are currently available in this issue, however, and most of them involved a limited number of patients and focused mainly on radiation-exposed populations.

**Materials and Methods:** We considered 59 pediatric patients who underwent surgery for DTC between 2000 and 2017. *RET/PTC* rearrangement was investigated with fluorescent *in situ* hybridization and real-time polymerase chain reaction. Sequencing was used to analyze mutations in the *BRAF, NRAS, PTEN, PIK3CA* genes, and the *TERT* promoter. The pediatric patients' clinical and molecular features were compared with those of 178 adult patients.

**Results:** In our pediatric sample, male gender and age <15 years coincided with more extensive disease and more frequent lymph node and distant metastases. Compared with adults, the pediatric patients were more likely to have lymph node and distant metastasis, and to need second treatments (*p* < 0.01). In all, 44% of the pediatric patients were found to carry molecular alterations. *RET/PTC* rearrangement was confirmed as the most frequent genetic alteration in childhood DTC (24.6%) and correlated with aggressive features. *BRAFV600E* was only identified in 16% of the pediatric DTCs, while NRASQ61R, NRASQ61K, and TERTC250T mutations were very rare.

**Conclusions:** Pediatric DTC is more aggressive at diagnosis and more likely to recur than its adult counterpart. Unlike the adult disease, point mutations have no key genetic role.

## Introduction

Thyroid carcinoma is the most common malignant neoplasm of the endocrine system. Papillary (PTC) and follicular (FTC) carcinomas are the most frequently seen histological types (accounting for more than 90% of cases) and belong to the family of differentiated carcinomas (DTC) originating from follicular thyroid cells (1).

Though less frequent than the adult type, DTC can also develop in pediatric age. There are significant clinical, pathological, and molecular differences between pediatric and adult patients, however. DTC in the pediatric population must consequently be characterized as a distinct entity with a separate diagnostic pathway and different treatment (2).

At the time of diagnosis, children are more likely than adults to present with advanced disease (3, 4). The incidence of distant metastases is estimated to be 25% in children, with lymph node involvement ranging from 40 to 80% in various studies (3, 5, 6). Positive lymph nodes and distant metastases from thyroid cancers are amenable to surgical resection and radioactive iodine (RAI) therapy, and these interventions have produced favorable outcomes in the pediatric patient population (5).

Our study aimed to characterize pediatric DTC in a large monocentric series of patients, most of them not exposed to radiation, focusing on: (i) clinical features and outcomes; (ii) molecular profile, with particular reference to the study of point mutations of the *BRAF, RAS, TERT* genes, and *RET/PTC* translocations; (iii) correlations between clinical and molecular findings; and (iv) comparisons between the clinical and molecular profile of pediatric DTC and that of a large series of adult thyroid carcinoma cases coming from our center.

## Materials and Methods

### Patients

Patients under 18 years of age with a histological diagnosis of DTC were retrospectively reviewed for this study, enrolling a series of 59 pediatric patients who underwent thyroid surgery between 2000 and 2017. Thirty of the Fifty-Nine patients (51%) also underwent neck dissection to remove the central/lateral lymph nodes.

All study samples were retrospectively selected from the electronic archives of the Surgical Pathology and Cytopathology Unit at Padua University. Clinical and histopathological data were obtained from electronic databases.

All patients underwent total thyroidectomy. Only information concerning their clinical presentation, pathological staging and histology was available for 8 patients; for the other 51, we had information concerning their clinical presentation, pathological staging and histology, local and systemic therapy, recurrences, subsequent treatments, and outcome. The follow-up was a mean 7 years (minimum 1, maximum 16), median 5.9 years.

After surgery, RAI ablation therapy was administered to 48/51 patients (94%), with a median dose of 100 mCi (range 30–200 mCi). Judging from their medical history, only 2 patients been exposed to radiation during childhood for the treatment of a previous tumor: one patient underwent fractioned total body irradiation (TBI) followed by allogenic bone marrow transplantation for acute lymphoblastic leukemia 11 years before the DTC development; the other patient underwent fractioned TBI plus chemotherapy followed by allogenic bone marrow transplantation for severe aplastic anemia 4 years before the DTC development.

The pediatric patients' clinical and molecular characteristics were compared with those of 178 consecutive adult patients who underwent surgery for PTC between 2007 and 2010, and were followed up at the Radiotherapy Unit and Endocrinology Unit in Padua. The TNM 7th edition (7) was applied to all patients to ensure a correct comparison of their pathological and clinical characteristics.

All studies were performed in accordance with the guidelines proposed in the Declaration of Helsinki: the local ethical committee (Ethical Committee for the Clinical Experimentation of the Hospital of Padua) approved our study protocol (Ref. 3388) and all patients (including the parent/guardian on behalf of the minor) gave their written informed consent.

### Outcomes

Patients were classified as being in remission if, at the time of their latest follow-up, their suppressed thyroglobulin (Tg) was <1 ug/L, Tg antibodies were negative, neck US was free of suspicious signs, and there were no pathological findings on any other imaging studies performed for clinically indicated reasons [whole body scan (WBS), radiography, computed tomography, 18-fluorodeoxyglucose (18FDG) positron emission tomography, or any other modality] or in any biopsy specimen. Patients with persistent disease at the time of their latest follow-up were classified as having either biochemically or structurally persistent disease, i.e., those with suppressed and/or stimulated Tg levels >1 ug/L but no structural evidence of disease were classified as having biochemically persistent disease, while those with structural evidence of disease (with or without abnormal Tg levels) were classified as having structurally persistent disease. Clinical status at final follow-up reflected not only the initial response to total thyroidectomy and RAI ablation therapy, but also the potential effects of continued levothyroxine suppressant therapy, any additional surgery or radiation therapy, and the passage of time.

### Molecular Analysis

#### BRAF, NRAS, PTEN, PIK3CA, and TERT Analysis

For all patients, DNA was extracted from formalin-fixed, paraffin-embedded (FFPE) tissues. The samples were deparaffinized as follows: 30 min in xylene; 15 min in 99% alcohol, 15 min in 95% alcohol; 15 min in 70% alcohol; 15 min in 50% alcohol; several washes in distilled water. DNA was extracted from the sample using the “DNeasy Blood and Tissue Handbook” extraction kit (Qiagen, Milano, Italy), according to the manufacturer's protocol. The *BRAF* gene (exon 5), *NRAS* (exon 3), *PTEN* (exons 5 and 8), *PIK3CA* (exons 9 and 20), and the *TERT* promoter were amplified using the polymerase chain reaction (PCR) technique. Mutation analyses were performed using direct sequencing ABI PRISM (Applied Biosystems, Foster City, California), as described elsewhere (8).

#### RET/PTC Rearrangement Analysis

*RET/PTC* translocations were investigated with both fluorescence *in situ* hybridization (FISH) and real-time polymerase chain reaction (RT-PCR).

To identify *RET/PTC* rearrangements (either 10q11.2 inversions or translocations), FISH was performed using the REPEAT-FREE POSEIDON RET (10q11) break-apart probe (Kreatech Diagnostics, Amsterdam, Netherlands) on FFPE samples. The FISH procedure was completed according to the Kreatech protocol with some modifications, as previously described (9). Slides were examined using an Olympus BIX-61 microscope (Olympus, Hamburg, Germany) with appropriate fluorescence excitation/emission filters. Signals were recorded by a CCD camera (Olympus Digital Camera). For microscopic examination, at least 100 intact and non-overlapping cell nuclei were scored for the presence of a split signal. Only cells with two overlapping signals, or one split and one overlapping signal, were counted to ensure that only complete cell nuclei had been scored. The signal pattern was interpreted as follows: interphase nuclei with two co-localized green/red fusion signals identified normal chromosomes 10, while separate red and green signals and green/red fusion signals indicated rearranged *RET*. To establish the cut-off for RET/PTC rearrangements, we performed a FISH analysis on ten samples of normal thyroid parenchyma and 100 nuclei were scored for the presence of a split signal. As previously reported, the cut-off was calculated as the mean +3 SD of *RET* rearranged cells (10).

For the RT-PCR analysis, tissue cores were deparaffinized with xylene at 50°C for 3 min. Total RNA extraction was done using the RecoverAll kit (Ambion, Austin, Texas, USA) according to the manufacturer's instructions. The nucleic acid extracted was analyzed with RT-PCR in an ABI-PRISM 7900HT Sequence Detector (Applied Biosystems, Milan, Italy) with the EntroGen Thyroid Cancer Mutation Analysis Panel Kit (EntroGen, Inc., Woodland Hills, California), which detects *RET/PTC1* (fusion between RET and CCDC6 genes), and *RET/PTC3* (fusion between RET and NCOA4 genes). Fusion detection reactions were performed with a 1-step procedure that combines complementary DNA synthesis and RT-PCR. The resulting RT-PCR amplification curves were visualized using Sequence Detection Software rel. 2.4 (Applied Biosystems).

### Statistical Analysis

The personal, clinical, and histopathological data were summarized using rates and percentages. All statistical analyses were performed using the MedCalc software (rel. 11.6.0). The distributions of the continuous variables were examined and the data were consequently summarized. The Mann-Whitney test and the *t*-test for independent samples were used to assess the differences in continuous variables (age at diagnosis, tumor size, and follow-up) between the two groups (pediatric vs. adult patients). The χ^2^-test was used to compare the variables within subgroups of the same series. Multivariate analysis was performed, using logistic regression, to confirm the independent role of different histopathological variables associated with final outcome. Differences were considered statistically significant when *p* was < 0.05.

## Results

### Clinical Features of Pediatric DTC Cases

The main clinical features of each pediatric patient are shown in Table 1.

**Table 1:** Clinic pathological features.

**Patient**	**Age at the diagnosis (years)**	**Gender**	**Tumor size (Mm)**	**Histological variant**	**T**	**N**	**M**	**Second treatment**	**Disease status**
1	17	F	8	cv-PTC	1a	X	X	0	BD
2	17	M	30	fv-PTC	2	0	0	0	Excellent
3	17	F	25	cv-PTC	2	1b	0	Surgery/RAI	Excellent
4	17	F	25	cv-PTC	3	0	0	n.a.	n.a.
5	17	M	10	cv-PTC	1a	1a	0	n.a.	n.a.
6	9	F	6	sv-PTC	1a	1a	0	0	Excellent
7	11	F	21	cv-PTC	3	1b	0	0	Excellent
8	17	F	22	cv-PTC	2	1a	0	Surgery	Excellent
9	18	F	13	cv-PTC	1b	X	0	n.a.	n.a.
10	18	F	12	cv-PTC	1b	1a	0	0	Excellent
11	16	F	24	cv-PTC	3	1b	0	RAI	BD
12	15	F	15	cv-PTC	3	1b	1	RAI	Excellent
13	17	F	25	cv-PTC	3	1b	0	0	Excellent
14	13	M	n.a.	PDTC	4a	1b	1	RAI/Surgery	BD
15	13	F	50	PDTC	3	1b	1	RAI	SD
16	8	M	20	cv-PTC	3	1b	1	RAI	SD
17	8	F	20	cv-PTC	3	0	0	n.a.	n.a.
18	16	F	12	cv-PTC	1b	1b	0	0	Excellent
19	17	F	3	cv-PTC	1a	X	0	n.a.	n.a.
20	14	M	12	cv-PTC	1b	1b	0	0	Excellent
21	15	M	35	FTC	2	X	1	0	Excellent
22	12	F	36	cv-PTC	3	1b	1	Surgery/RAI	SD
23	17	F	18	cv-PTC	3	1a	0	0	Excellent
24	17	F	13	cv-PTC	3	1b	0	0	Excellent
25	9	M	42	cv-PTC	4a	1	1	RAI	SD
26	14	F	40	cv-PTC	3	1a	0	Surgery	BD
27	13	F	11	cv-PTC	3	1a	0	Surgery	Excellent
28	14	F	20	cv-PTC	3	1b	0	RAI	SD (Neck)
29	9	F	35	cv-PTC	3	1b	0	0	Excellent
30	12	M	22	cv-PTC	4a	1a	1	Surgery/RAI	SD
31	14	F	3	cv-PTC	1a	1	0	0	Excellent
32	14	M	10	cv-PTC	3	0	0	Surgery/RAI	BD
33	17	F	11	cv-PTC	3	0	0	0	Excellent
34	17	F	35	fv-PTC	2	0	0	0	Excellent
35	11	F	20	cv-PTC	3	1a	0	0	Excellent
36	14	F	30	cv-PTC	3	1b	0	0	Excellent
37	15	F	12	cv-PTC	3	1a	0	Surgery	Indeterminate
38	17	F	25	fv-PTC	2	0	0	0	Excellent
39	15	F	15	cv_PTC	1b	0	0	0	Excellent
40	15	F	12	fv-PTC	1b	0	0	0	n.a.
41	17	M	20	FTC	4	1	1	RAI	Excellent
42	6	F	15	fv-PTC	1b	0	0	n.a.	n.a.
43	13	M	n.a.	sv-PTC	3	1b	0	0	Excellent
44	18	F	22	cv-PTC	3	1b	0	0	Excellent
45	16	M	38	cv-PTC	2	0	0	0	Excellent
46	17	F	7	fv-PTC	1a	0	0	n.a.	n.a.
47	13	F	25	fv-PTC	3	1a	0	0	Excellent
48	16	F	7	cv-PTC	1a	0	0	0	BD
49	16	F	15	cv-PTC	1b	0	0	0	Excellent
50	17	F	8	cv-PTC	1a	1a	0	Surgery	BD
51	12	M	40	cv-PTC	4a	1b	0	0	BD
52	16	F	11	cv-PTC	1b	1a	0	0	Excellent
53	15	M	12	cv-PTC	1b	1a	0	0	Excellent
54	15	F	58	cv-PTC	3	1b	0	0	Excellent
55	17	M	35	cv-PTC	3	1b	0	0	SD (Neck)
56	11	F	n.a.	sv-PTC	4a	1b	1	RAI	SD
57	10	F	35	sv-PTC	3	1a	X	0	Excellent
58	17	M	19	tcv-PTC	1b	1a	0	0	Excellent
59	15	F	25	FTC	2	0	0	0	Excellent

*M, male; F, female; n.a., not assessable/not available; cv-PTC, classical variant of papillary thyroid carcinoma; fv-PTC, follicular variant of PTC; sv-PTC, sclerosing diffuse variant of PTC; tcv-PTC, tall cell variant of PTC; FTC, follicular thyroid carcinoma; PDTC, poorly differentiated carcinoma; RAI, Radioactive Iodine; BD, biochemical disease; SD, structural disease*.

Average age at diagnosis was 14.4 ± 2.9 years (range 6.4–17.8 years): 29/59 patients (49%) were <15 years old (defined as “children”), and 30/59 (51%) were ≥15 (defined as “adolescents”). There were 16/59 males (27%), and 43/59 females (73%), with a male/female ratio of 1:2.7.

At histological analysis, 42/59 (71.2%) cases were the classic variant of PTC (cv-PTC), 7/59 (11.8%) were the follicular variant of PTC (fv-PTC), 4/59 (6.8%) were the sclerosing variant of PTC (sv-PTC), 1/59 (1.7%) was the tall cell variant of PTC (tcv-PTC), 3/59 (5.1%) were cases of follicular thyroid carcinoma (FTC), and 2/59 (3.4%) were poorly-differentiated thyroid carcinomas (PDTC).

Data on tumor size were available for 56/59 (95%) patients (no clear definition of tumor size was provided for 3 patients). The median size of the primary tumor was 20.0 mm (range 3–58 mm): 9/56 tumors (16.0%) were ≤10 mm (microcarcinomas), and 47/56 (84.0%) were >10 mm in size. As suggested by Nikita et al., the lesions' size was further classified as follows: 26/56 (46.4%) were <20 mm; 27/56 (48.2%) were 20–40 mm; and 3/56 (5.4%) were >40 mm (11).

Multifocality was detected in 25/59 patients (42.4%), extra-thyroid extension in 25/59 (42%), and vascular invasion in 48/59 (81.4%). There were lymph node metastases in the lateral compartment in 20/59 patients (33.9%). Distant metastases were identified on WBS in 10/48 patients who underwent RAI: the site involved was the lung in 9/10 and the mediastinum in 1/10.

No significant differences in tumor size emerged between the male and female patients (with a median tumor size of 24.6 mm and 20.1 mm, respectively; *p* = 0.23). The extent of the disease (T) was gender-related, however: 10/16 males (63%) had T3 or T4 disease, while the proportion among females was 22/43 (51%; *p* < 0.01). Gender was also significantly associated with the presence of distant metastases, and therefore with the most advanced stage: 6/16 (38%) males had metastatic disease, as opposed to only 4/43 (9%) females (*p* = 0.03). No significant associations came to light between histotype, multifocality, lymph node involvement, need for second treatment, or final disease status.

Table 2 shows the main pathological and clinical characteristics correlated with the presence/absence of metastases for all 59 patients. The 8 patients for which we had no information about their treatment and follow-up were included because they started with a low-risk disease. They were considered as M0-x.

**Table 2:** Correlation with clinic-pathological features and presence of distant metastasis at the diagnosis.

	**Total (59)**	**M1 (10)**	**M0-x (49)**	***p*-value**
Gender				**0.03**
M	16/59 (27%)	6/16 (37.5%)	10/16 (62.5%)	
F	43/59 (73%)	4/43 (9.3%)	39/43 (90.7%)	
Age (average)	14.40	12.50 ± 2.75	14.89 ± 2.44	**0.01**
Tumor size (median; mm)	20.0	30	21	0.08
T				**0.0007**
1	19/59 (21.7%)	0/19 (0%)	19/19 (100%)	
2	8/59 (13.6%)	1/8 (12.5%)	7/8 (87.5%)	
3	26/59 (44.1%)	4/26 (15.4%)	22/26 (84.6%)	
4	6/59 (10.2%)	5/6 (83.3%)	1/6 (16.7%)	
Extrathyroidal extension				0.10
Yes	25/59 (42.3%)	8/25 (32.0%)	17/25 (68%)	
No	34/59 (57.7%)	2/34 (5.9%)	32/34 (94.1%)	
Multifocality				0.13
Yes	25/59 (42.3%)	7/25 (28%)	18/25 (72%)	
No	34/59 (57.7%)	3/34 (8.8%)	31/34 (91.1%)	
Vascular invasion				0.28
Yes	48/59 (71.2%)	10/48 (20.8%)	38/48 (79.2%)	
No	11/59 (6.7%)	0/11 (0%)	11/11 (100%)	
Lymph nodal metastasis				**0.02**
N0 and N1a	30/59 (50.8%)	2/30 (6.7%)	28/30 (93.3%)	
N1b	20/59 (33.9%)	7/20 (35.0%)	13/20 (65%)	
Nx	9/59 (15.3%)	1/9 (11.1%)	8/9 (88.9%)	

*F, female; M, male*.

*Bold values are the statistically significant associations*.

The presence of distant metastases was found significantly associated with younger age (*p* = 0.01), larger and more extensive tumors (*p* < 0.01), and cervical lymph node metastases (*p* = 0.02).

On multivariate analysis, only T4 status was identified as an independent predictor of distant metastases (OR 43.75, CI: 4.04 to 474.27).

During the follow-up, 18/51 (35.3%) patients received further treatments, including: second surgery in 5/18 (27.8%) patients; RAI in 8/18 (44.4%), and both in 5/18 (27.8%). Positive lymph nodes and distant metastases were associated with a higher likelihood of undergoing a second treatment (*p* = 0.04 and *p* = 0.002, respectively).

Among the 51 patients with follow-up data, the final state of the disease was remission in 34/51 (66.7%) cases, persistent disease in 16/51 (31.3%; 8 with biochemically evident disease, and 8 with structurally evident disease); and for one patient who recently had surgery for a recurrence in the lateral neck the final status was classified as undetermined (restaging is ongoing).

Of the 8 patients with a final status of structurally evident disease, 6 had distant metastases at: 5/6 had stable lung disease, and 1 had progression in the lung with lesions failing to uptake RAI or 18-FDG. The other 2 patients with structurally evident disease had a local progression and are currently being reassessed for further surgery.

**Figure 1:**
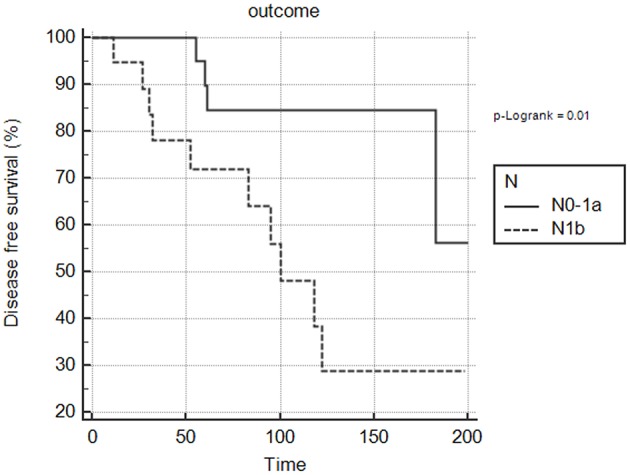
Correlation between lateral cervical (N1b) lymph node metastases and disease status in pediatric population. The Kaplan-Meier curve shows N1b significantly associated with persistent/recurrent disease at the end of the follow-up (*p* = 0.01).

Disease status at the end of the follow-up correlated with disease stage: 9/41 patients in stage I (22%) had persistent/recurrent disease (biochemically evident in 7, and structurally evident in 2), while 7/10 (70%) patients in stage II had persistent/recurrent disease (biochemically evident in 1 and structurally evident in the lung in 6) (*p* = 0.002). There was also a significant association between disease status and the need for a second treatment: among patients who underwent a second treatment, 12/18 (66.7%) had persistent/recurrent disease (5 biochemically evident and 7 structurally evident); in the group of patients given no second treatments, 4/33 (12.1%) had persistent/recurrent disease (3 biochemically evident, 1 with a structurally evident neck progression being assessed for further surgery) (*p* = 0.0001). The final outcome also correlated significantly with tumor size, T stage, cervical lymph node and distant metastases (*p* < 0.05) (Table 3). Kaplan-Meier curves showed that patients with lymph node metastases in lateral neck compartments more frequently had persistent/recurrent disease (*p* = 0.01) (Figure 1).

**Table 3:** Correlation with clinic-pathological features and outcome.

	**Total (51)**	**Structural disease (8/51)**	**Biochemical disease (8/51)**	**Indeterminate (1/51)**	**Remission (34/51)**	***P*-value**
Tumor size (mm)	21.5	32.1	19.5	12	21.5	0.10
T						0.05
1	12 (23.5%)	0/12 (0%)	3/12 (25%)	0/12 (0%)	9/12 (75%)	
2	9 (17.6%)	0/9 (11.1%)	0/9 (0%)	0/9 (0%)	9/9 (100%)	
3	24 (47.1%)	5/24 (20.8%)	3/24 (12.5%)	1/24 (4.2%)	15/24 (62.5%)	
4	6 (11.8%)	3/6 (50%)	2/6 (33.3%)	0/6 (0%)	1/6 (16.7%)	
Lymph nodal metastasis						**0.01**
N0–N1a	30 (58.8%)	1/30 (3.3%)	4/30 (13.4%)	1/30 (3.3%)	24/30 (80%)	
N1b	19 (37.3%)	7/19 (36.8%)	3/18 (15.8%)	0/19 (0%)	9/19 (47.4%)	
Distant metastasis						**0.0008**
M1	10 (19.6%)	6/10 (60%)	1/10 (10%)	0/10 (0%)	3/10 (30%)	
TNM staging						
I	41 (80.4%)	2/41 (4.9%)	7/41 (17.1%)	1/41 (2.4%)	31/41 (75.6%)	**0.0003**
II	10 (19.6%)	6/10 (60%)	1/10 (10%)	0/10 (0%)	3/10 (30%)	
Second treatment	18 (35.3%)	7/18 (38.9)	5/18 (27.8%)	1/18 (5.6%)	5/18 (27.8%)	**0.0001**

*Bold values are the statistically significant associations*.

One multivariate analysis, only the presence of distant metastases independently correlated with persistent disease (OR 13, 95% CI 2.19 to 77.03).

### Molecular Characterization

In all, 26/59 patients (44%) of our series were carriers of molecular alterations.

#### Point Mutations

Mutually-exclusive point mutations were found in 13/50 (26%) tissue samples from which the genetic material could be amplified. No point mutations were documented in patients under 10 years old, while there was 1 among 13 (8%) 10- to 14-year-olds, and 12 among 13 (92%) 15- to 18-year-olds.

Among the samples that could be tested for the presence of *BRAF* mutations, the V600E mutation was found in 8/50 (16%) cases. All mutations belonged to samples of cv-PTC (7/8) and tcv-PTC (1/8). No other *BRAF* mutations were identified.

Among the samples that were examined for the presence of *NRAS* mutations, a total of 4/52 (8%) molecular events came to light. The Q61K mutation was found in 2 samples and the Q61R mutation in other 2. All mutations belonged to samples of fv-PTC. Among the samples tested for the presence of *TERT* promoter mutations, the C250T mutation was only found in one PTC sample (1/44).

Among the samples successfully amplified for PCR, no mutations were identified in exons 5 or 8 of PTEN, or in exons 9 or 20 of PIK3CA.

#### RET/PTC Rearrangements

**Figure 2:**
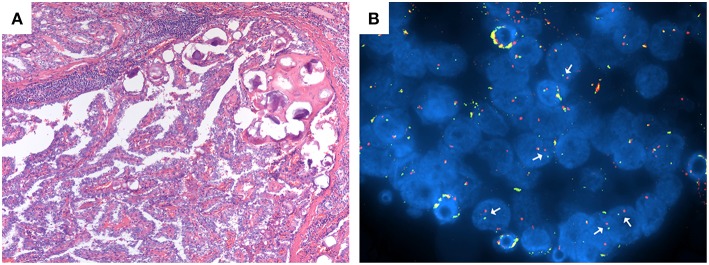
**(A,B)** Patient n. 37: classical variant PTC at histological analysis with papillary structure and psammoma bodies (**A**; magnification 100x) and RET/PTC translocation at FISH analysis **(B)**.

Among the samples that could be tested for the presence of *RET/PTC* rearrangements, 14/57 (24.6%) cases were found to carry the translocation (Figure 2). RT-PCR data analysis confirmed the results obtained with the FISH method. Interestingly, 1 of these 14 patients had both the *RET/PTC* translocation and a *TERT* promoter mutation and was classified as a case of PDTC.

#### Correlation Between Clinical and Molecular Features

*BRAF*V600E was related to the need for a second treatment: 4/6 *BRAF*V600E-mutated patients (66.7%) underwent a second treatment, as opposed to only 8/35 (22.9%) in the group of patients without this mutation (*p* = 0.04). No information on any second treatment or follow-up were available for 2/8 *BRAF*V600E-mutated patients. Two of the 8 *BRAF*V600E-mutated patients (25%) were <15 years old, while 6/8 (75%) were ≥15–18 years old (*p* = 0.11) (Table 4). No significant association was found between *BRAF*V600E mutation status and sex, tumor size, histotype, multifocality, lymph node and distant metastasis, stage, or disease outcome.

**Table 4:** Comparison of clinical and pathological features and outcomes between children and adolescents with DTC and between pediatric and adult patients.

	**Children (*n* = 29)**	**Adolescents (*n* = 30)**	***p*-Value**	**Pediatric patients (*n* = 59)**	**Adult patient (*n* = 178)**	***p*- Value**
Gender F/M	19/10 (1.9:1)	24/6 (4:1)	0.215	43/16 (2.7:1)	138/40 (3.5:1)	0.46
T			**0.009**			**0.01**
1	4/29 (13.8%)	14/30 (43%)		18/59 (30.4%)	68/177 (75.7%)	
2	3/29 (10.3%)	2/30 (20%)		9/59 (15.3%)	18/177 (10.2%)	
3	17/29 (58.6%)	9/30 (30%)		26/59 (44.1%)	88/177 (49.7%)	
4	5/29 (17.2%)	1/30 (3.3%)		6/59 (10.2%)	3/177 (1.7%)	
Lymph nodal metastasis	24/29 (82.6%)	16/30 (53.3%)	**0.03**	40/59 (67.8%)	75/178 (42.1%)	**0.0004**
Distant metastasis	9/29 (31%)	1/30 (3.3%)	**0.009**	10/59 (16.9%)	7/178 (3.9%)	**0.0003**
Second treatment	13/29 (44.8%)	5/25 (16.7%)	**0.03**	18/51 (35.3%)	18/178 (10.1%)	**<0.0001**
Disease Status			**0.02**			**<0.0001**
Remission	15/27 (55.6%)	19/24 (79.2%)		34/51 (66.7%)	157/176 (89.2%)	
Biochemical disease	4/27 (14.8%)	4/24 (16.7%)		16/51 (31.4%)^*^	14/176 (8%)^*^	
Structural disease	7/27 (25.9%)	1/24 (4.2%)				
Indeterminate	1/27 (3.7%)	0/24 (0%)		1/51 (8%)	1/176 (0.6%)	
Death	0/27 (0%)	0/24 (0%)		0/51 (1.9%)	4/176 (2.3%)	

*F, female; M, male*.

* *In the comparison of final outcome between pediatric and adult population, biochemical and structural disease are considered together as persistence/recurrent disease*.

*Bold values are the statistically significant associations*.

*NRAS* mutations were associated with histology: 4/4 samples were fv-PTC. Significant associations were found between *NRAS* mutations and the absence of vascular invasion or lymph node metastases.

The only patient carrying the *TERT* promoter mutation (with a concomitant *RET/PTC* rearrangement, as mentioned above) had a locally advanced PDTC with cervical lymph node metastases (N1b) and distant metastases. At the end of the follow up (46 months) the patient had persistent disease.

As mentioned earlier, *RET/PTC* rearrangements were found in 14 patients: 5/14 were *RET/PTC* 3 (35.7%), and 9/14 (64.8%) were *RET/PTC* 1. The two patients with a history of exposure to radiation carried this translocation (1 with *RET/PTC* 1, the other with *RET/PTC* 3). As for histotype, 2 patients were cases of PDTC, 10 were cv-PTC, and 2 were sv-PTC. *RET/PTC* rearrangement was found significantly correlated with aggressive features such as lymph node metastases (*p* = 0.01), and *RET/PTC* 3, in particular, correlated with both cervical lymph node involvement (N1b) and distant metastases (*p* = 0.02 and *p* = 0.03, respectively). Moreover, 4/5 patients with *RET/PTC* 3 rearrangements needed a second treatment during their follow-up (*p* = 0.03). As for the 8 patients with a final status of structurally evident disease, 1 was a young female with lung progression whose lesions showed no uptake of RAI or 18-FDG, and she had the *RET/PTC 3* translocation.

#### The Clinical and Molecular Profile of Pediatric vs. Adult Thyroid Cancer

The main clinical, pathological and histopathological characteristics and outcomes of the samples of pediatric (children and adolescents) and adult patients are summarized in Table 4.

A more advanced disease at diagnosis was apparent in the younger patients: 22/29 patients <15 years old (75.8%) had T3 or T4 tumors, as opposed to 10/30 (33.3%) older pediatric patients (*p* < 0.01). Lymph node metastases were also more common in the former (24/28; 85.7%) than in the latter (14/27; 51.9%, *p* = 0.007). Of the 10 patients with distant metastases, 8 were <15 years old (*p* = 0.01). These two subgroups showed no significant differences in terms of primary tumor size, multifocality, histotype, need for second treatment, or disease status at the end of the follow-up, though only 56% of the younger group vs. 81% of the older one reached disease remission at the end of the follow-up.

In the adult population, 138/178 (77.5%) patients were female and 40/178 (22.5%) were male, with a male: female ratio of 1:3.5. The sex distribution was homogeneous between the pediatric and adult groups (*p* = 0.46). The average age of the adults was 48.6 ± 13.1 years (range 22–81 years).

The pediatric patients had larger lesions than the adults (median 23.6 vs. 19.3 mm); only 26/56 (46.4%) of the former, as opposed to 134/177 (75.7%) of the latter had a tumor <20 mm in diameter (*p* = 0.0004).

Lymph node metastases were found in 40/59 pediatric patients (67.8%) and 75/178 adults (42.1%) (*p* < 0.001). Distant metastases were more common in the pediatric patients too, involving 10/51 (19.6%) as opposed to 7/178 adults (3.9%) (*p* < 0.001). All distant metastases in both groups involved the lung, with the sole exception of a 17-year-old male with mediastinal disease.

The children and adolescents were significantly more likely to have a second treatment: 18/51 pediatric patients (35.3%) and 18/178 adults (10.1%) had further surgery or radiometabolic therapy (*p* < 0.0001). Age was found associated with final disease status: 34/51 pediatric patients (66.7%) and 157/176 adults (89.2%) were in remission (*p* < 0.0001). Among the adult patients, 4/176 (2.3%) died of their disease, while no pediatric patients died of cancer-related causes.

As regards the molecular profile, the rate of *BRAF*V600E mutations was significantly higher in adult PTC than in pediatric PTC, with 107/178 (60%) and 8/50 (16%), respectively (*p* < 0.0001). Although *NRAS* mutations emerged in 8% of the pediatric patients (4/52) as opposed to 2.2% (4/178) of the adults, this difference was not statistically significant.

## Discussion

DTC is rare in the pediatric population and its clinical, pathological and molecular characteristics differ from those of adult DTC (12). In children and adolescents, it has a more aggressive presentation at diagnosis, and a higher frequency of lymph node and distant metastases (13–18). There is also a greater risk of disease recurrence in the pediatric population. The prognosis is nonetheless excellent and the mortality rate is very low (2, 19).

Some Authors argue that the clinical and pathological differences between pediatric and adult DTC are due to a different genetic profile (20). The most common molecular event in children and adolescents (particularly after exposure to radiation) is represented by *RET/PTC* rearrangements, while point mutations in *BRAF, RAS* or *TERT* promoter genes are the main genetic drivers of tumorigenesis in adults (21). Somatic mutations (which are infrequent in pediatric age) are usually associated with cellular dedifferentiation, genetic instability, and a reduced NIS iodine transporter expression. The paradox of pediatric DTC—with its worse presentation at diagnosis, but good outcome—could therefore be explained by a greater differentiation of the tumor cells and a consequently better response to radiometabolic treatments and TSH suppression therapy (22, 23) and also by a lower frequency of *BRAF* mutations (24) Regarding the potential effects of continued levothyroxine suppression, TSH contributes to the regulation of thyrocyte differentiation by modulating thyroid gene levels (25).

The clinical and histological features seen in our pediatric population are similar to those already described in other studies. The majority of our patients were female, with males and younger patients presenting with more advanced disease. Male gender coincides with more aggressive tumor features in terms of the extent of the primary tumor at diagnosis, dissemination outside the thyroid and to distant sites. On the other hand, males did not have significantly larger tumors, nor did they differ from females in terms of final outcome (26–28). Younger patients had a more aggressive tumor at diagnosis in terms of extent, lymph node involvement and distant metastases than older pediatric patients although, here again, age did not affect final outcome in our sample. In addition, the prevalence of DTC was higher among females in both subgroups, but the female/male ratio was higher for the older than for the younger pediatric patients, although the difference was not statistically significant (4:1 vs. 1.9:1). This trend is in line with findings in adult DTC, and could be influenced by hormonal aspects, and the impact of estrogens in particular. These data seem to suggest that DTC in childhood is locally more aggressive and should be considered as a distinct clinical entity from DTC in adolescence.

In the literature, the frequency of lymph node metastases in the pediatric population varies between 50 and 75%, while for distant metastases it ranges between 6 and 33% (5, 17, 19, 29, 30). Our findings confirm this aggressiveness at diagnosis, with a prevalence of 67.7% for lymph node positivity and 19.6% for distant metastases. In our series, DTC dissemination outside the thyroid gland emerged as the main factor associated with the risk of distant metastases.

As expected, lymph node and distant metastases were significantly associated with a higher chance of undergoing further treatment during the follow-up.

The optimal management of neck disease depends on several factors, including the size and site of disease, previous treatments, disease progression rate, and the patient's age. Some patients in our sample known to have persistent disease were treated months or even years afterwards: 9 underwent second surgery, with or without further RAI, and all except one of them achieved at least a structurally evident remission.

Distant metastases in pediatric cases usually involve micro-nodular lung lesions with an excellent RAI uptake, characteristics that can explain why distant metastases in children are more amenable and responsive to RAI therapy than those in adults. There were 10 young patients with lung metastases in our sample and, after one or more RAI treatments, 3 obtained a complete remission, 1 had only biochemically evident disease, and 6 had persistent lung metastases, with only one patient in progression. Our multivariate analysis indicated that only the presence of distant metastases independently correlated with persistent disease (OR 13.95 % CI 2.19 to 77.03).

As emphasized by recent ATA guidelines (2), our data highlight the importance of a tailored assessment of children and adolescents with DTC. The aim should be to identify already at the initial diagnosis which pediatric patients would benefit from more aggressive surgical and radiometabolic approaches, and a closer follow-up. Clinical status at final follow-up reflects not only initial response to total thyroidectomy and RAI ablation, but also the potential effects of continued levothyroxine suppression and/or further surgery or RAI therapy over time.

Comparing our cohort of pediatric patients with an adult PTC population treated and followed up at the same center confirmed the greater aggressiveness at diagnosis of pediatric DTC. Tumor size was significantly larger in patients <18 years old, and there was a greater frequency of lymph node metastases (67.7 vs. 47.5%) and distant metastases (19.6 vs. 3.9%) than in adults. In terms of survival, however, the prognosis for pediatric DTC is excellent, and better than for the adult counterpart.

As for the molecular aspects, pediatric patients and adults clearly showed a different genetic profile. Even though almost all of the patients in our series had no history of exposure to radiation, *RET/PTC* rearrangements were confirmed as the most common genetic alteration in pediatric DTC (24.6%). *RET/PTC* rearrangements, and *RET/PTC* 3 in particular, were found to correlate with aggressive clinical and pathological features such as lymph node and distant metastases. Patients with the *RET/PTC* 3 translocation needed more second treatments (*p* = 0.03), suggesting [as reported in the literature (31)] that this molecular event results in a lower response to RAI and a consequent need for further treatments to achieve disease remission. In fact, the only patient with progressive lung disease in our series who became radio-refractory carries this translocation.

The *BRAFV600E* mutation is the most common in adult PTC (32–35), while in pediatric patients its prevalence varies between 0 and 37% (20). Our study is in line with previous reports: this mutation was found in 16% of our pediatric patients and 60.1% of our adult sample. Although the association was not statistically significant, we found that the frequency of *BRAF* mutations increased with age at diagnosis, even in the pediatric population, as seen in previous studies: it was 0% in patients under 10 years old, 14% among those between 11 and 14 years old, and 86% among the 15- to 18-yea-olds (21, 36). Several studies identified an association in adult DTC between *BRAF* mutations and more aggressive clinical, pathological and histopathological features, more recurrences and a higher mortality. This relationship has not been seen in pediatric populations (35, 36).

Our study also found no significant associations between *BRAF* mutations and gender, tumor size, histotype, multifocality, lymph node metastases, stage of disease or final outcome. The presence of a *BRAFV600E* mutation nonetheless correlated significantly with the need for a second treatment during the follow-up: among the *BRAFV600E*-positive patients, 4/6 (67%) received further treatment, as opposed to 11/42 (26%) in the group of patients without this mutation (*p* = 0.04). This finding prompts us to speculate that, as seen in adults, *BRAF* mutations may be associated with more aggressive clinical features and a higher risk of recurrence or persistence of disease in the pediatric population too.

Mutations in the *RAS* gene are rare in pediatric patients with DTC, the prevalence found in various studies to range between 0 and 7% (20). The rarity of this molecular event was confirmed in our study too: only 8% of pediatric patients had a *NRAS* mutation. In adults, *RAS* is more frequently mutated in cases of FTC (~40%), and fv-PTC (~15–20%) (37). The association between *RAS* gene alterations and the fv-PTC histology was confirmed in our pediatric series as well. In the adult control group, the frequency of *NRAS* gene mutations was 3%, i.e., lower than reported in the literature—a discrepancy probably due to the fact that only 6% of PTCs in our adult population were the follicular variant. In our pediatric population, some significant associations emerged between *NRAS* gene mutations and the clinical and pathological features of the DTC. *NRAS*-mutated DTC correlated with older age, no vascular invasion, and no lymph node or distant metastases. These data suggest that *NRAS* mutations do not have a key role in the pathogenesis of pediatric DTC, and might be a hallmark of a subset of less aggressive tumors, as seen in the albeit limited number of cases presenting this mutation in other studies (11, 18, 38).

*TERT* promoter, *PTEN*, and *PIK3CA* gene mutations are rare in adult DTC, while they have been found more frequently in poorly-differentiated carcinomas and anaplastic carcinoma (39–42). In pediatric age, though few studies have been conducted, the prevalence is even lower (1%) (38). This is consistent with the observation of a greater differentiation of tumor cells in pediatric DTC.

Interestingly, our one pediatric patient carrying a *TERT* mutation had advanced-stage disease at diagnosis, multifocality, bilateral extra-thyroid extension, areas of poorly-differentiated carcinoma, and lymph node and distant metastases. He also had a concomitant *RET/PTC* translocation. The boy underwent second treatments involving both surgery and RAI, achieving a final disease status of biochemically persistent disease. This picture is consistent with previous reports in adult series: in thyroid carcinoma, *TERT* promoter mutations are associated with more aggressive histopathological features and a worse prognosis (40).

In conclusion, our study showed that DTC in pediatric age has different clinical, pathological and prognostic features from its counterpart in adults: it is more aggressive at diagnosis and carries a greater risk of persistence/recurrence. Within the pediatric group, special attention should be paid to male patients under 15 years old, as they are associated with a more advanced disease at diagnosis, although their final disease status does not seem to be affected by gender or age. As for the molecular profile, there are substantial differences between pediatric and adult DTC. *RET/PTC* translocations are the main molecular event in the pediatric population, while *BRAF*V600E mutations are significantly less common in pediatric DTC than in adults, and they are unassociated with the clinical and histopathological features of the disease. Finally, mutations in the *TERT* promoter and *NRAS, PTEN*, and *PIK3CA* genes are occasional molecular drivers of cancer in the pediatric population. Unlike the case in adults, point mutations do not have a key genetic role in children, even those not exposed to ionizing radiation.

## Data Availability

The raw data supporting the conclusions of this manuscript will be made available by the authors, without undue reservation, to any qualified researcher.

## Ethics Statement

This study was carried out in accordance with the recommendations of Padova Hospital Ethical Committee protocol No. 58403 with written informed consent from all subjects. All subjects gave written informed consent in accordance with the Declaration of Helsinki.

## Author Contributions

FG and FV: study concept and design, analysis and interpretation, drafting of the manuscript, and final approval of the version to be published. CM and GP: study concept and design, supervision, and final approval of the version to be published and agreement with all aspects of the work. SCe, SB, LB, SW, MR, MI, and SCa: substantial contributions to data acquisition and interpretation, critical revision of the manuscript, and final approval of the version to be published. JM: substantial contributions to data acquisition. CC: analysis and interpretation, critical revision of the manuscript, final approval of the version to be published, and agreement with all aspects of the work.

### Conflict of Interest Statement

The authors declare that the research was conducted in the absence of any commercial or financial relationships that could be construed as a potential conflict of interest.
